# Optimization of the Microwave-Assisted Extraction Process of Bioactive Compounds from Annatto Seeds (*Bixa orellana* L.)

**DOI:** 10.3390/antiox8020037

**Published:** 2019-02-06

**Authors:** Julian Quintero Quiroz, Angélica Celis Torres, Luisa Muñoz Ramirez, Mariluz Silva Garcia, Gelmy Ciro Gomez, John Rojas Camargo

**Affiliations:** 1 College of Pharmaceutical and Food Sciences, University of Antioquia, Calle 67 No. 53-108, University Campus, Medellín 050010, Colombia; angelica.celis@udea.edu.co (A.C.T.); luisa.munozr@udea.edu.co (L.M.R.); gelmy.ciro@udea.edu.co (G.C.G.); jhon.rojas@udea.edu.co (J.R.C.); 2 Institute of Food Science and Technology (INTAL), Cra. 50g #12S-91, Itagüi 055412, Colombia; marisylva87@gmail.com

**Keywords:** antimicrobial activity, antioxidant activity, polyphenol compounds, bixin

## Abstract

This study deals with the extraction, optimization, and evaluation of the antioxidant and antimicrobial activities of bioactive compounds obtained from the seeds of annatto using microwave-assisted extraction as compared to leaching. Annatto seeds were subjected to a microwave treatment of 2450 MHz and power of 700 watts using a response surface design involving four factors: pH (4–11), solvent concentration (ethanol) (50–96%), solvent-to-seed ratio (2–10), and microwave exposure time (0–5 min). The contents of polyphenol compounds and bixin were taken as response variables. Subsequently, the antioxidant and antimicrobial activities were assessed at the optimal processing conditions predicted by the experimental design. Microwaves, solvent concentration, and the solvent-to-seed ratio showed a statistically significant effect for the extraction of polyphenol compounds and bixin. Thus, microwaves accelerated the extraction of those compounds and the slight increase in temperature caused some degradation of the polyphenol compounds. The microwave-assisted extraction increased the contents of polyphenols and bixin along with their antioxidant activity as compared to leaching extraction. However, this technique does not significantly improve the antimicrobial activity against *Bacillus cereus* and *Staphylococcus aureus*.

## 1. Introduction

The reddish or orange extract obtained from the seeds of annatto (*Bixa orellana* L.) has a great coloring strength, making this extract useful in the food and cosmetic sectors. In addition, the extract possesses inflammatory, antioxidant, and antimicrobial properties [1,2]. These pharmacological activities are due to diverse bioactive compounds such as polyphenols (i.e., hypolatin and caffeic acid) and carotenoids (i.e., bixin or 6-methyl hydrogen (9Z)-6,6′-diapocarotene-6) [3]. The antioxidant activity of these compounds is attributed to their high structural conjugation and capability to couple with singlet oxygen which is responsible for denaturation of proteins belonging to cell membranes, leading to cell lysis [1,4,5]. 

Recent reports deal with the biological activity of annatto extract obtained by traditional techniques, which are compelling for the pharmaceutical and food companies. For instance, Zhang and collaborators assessed the ability of bixin to promote transcription of a new Ets-related factor (Nrf2) and E74-like factor 2 (ELF2) in a murine model with inflammation in the lung tissue due to SiO_2_ particles. This transcription factor regulates the inducible expression of numerous genes of detoxifying enzymes and antioxidants by binding to a specific DNA sequence known as the antioxidant response element (ARE), resulting in protection against several pathologies such as cancer, liver toxicity, and inflammation [6]. Further, the activation of Nrf2 in mice potentiates the antioxidant and healing abilities and decreased lung tissue inflammation after inhalation of bixin particles [2]. On the other hand, Viuda and collaborators determined the in vitro antioxidant and antimicrobial activities of annatto seed extract. They used five different hydrogen-donating or radical-scavenging tests including the 2,2′-diphenyl-1-picrylhydrazyl (DPPH) stable radical, ferric-reducing antioxidant power (FRAP), thiobarbituric acid reactive substance (TBARS), Rancimat, and ferrous ion (Fe^2+^) chelating activity. On the other hand, the microdilution in broth method was used to evaluate the antimicrobial activity against *Listeria innocua* (Spanish Type Culture Collection, University of Valencia, Research Building, Burjassot, Spain. (CECT) 910), *Aeromonas hydrophila* (CECT 5734), *Bacillus cereus* (American Type Culture Collection (ATCC) 11778), and *Pseudomona aeruginosa* (ATCC 9027). They found the annatto seed extract as an alternative preservative to replace the synthetic ones such as butyl-hydroxy toluene (BHT) in food matrices due to its extensive antioxidant activity (93.01 ± 1.22, 75.88 ± 0.21, 25.41 ± 3.52, 10.53 ± 0.31, and 1.34 ± 0.07 for DPPH (inhibition, %), TBARS (inhibition, %), FRAP (TEAC), FIC (chelating effect, %), and rancimat (AAI), respectively. Conversely, it had a mild antimicrobial action compared to some synthetic antimicrobials (1.024 mg/mL, 256 mg/mL, 512 mg/mL, and 256 mg/mL for *P. aeruginosa, B. cereus, L. innocua,* and *A. hydrophila*, respectively) [1].

Maceration, leaching, extraction with supercritical fluids (CO_2_), dispersive liquid–liquid, sonication, enzymatic extraction, microextraction, and microwave-assisted extraction (MAE) are the most widely used methods for the extraction of bioactive molecules from plants [7]. However, the extraction technique affects the functional activities of the extract and thus, conventional approaches require long processing times, exposing the active compounds to degradation and resulting in low efficiency. On the other hand, some emerging extraction techniques such as supercritical CO_2_ extraction are costly and involve high energy consumption [8]. Further, the use of mechanical methods, spouted bed, sonication, microwaves, and supercritical fluids (CO_2_) as emerging extraction technologies, have been focused simply on the extraction of bixin and norbixin [7,9,10,11]. Most of these studies highlight the coloring capacity of the extract, and others evaluate the effect of the extraction technique on the antioxidant and antimicrobial activities separately [11,12].

The microwave-assisted extraction (MAE) involves the formation of high-energy electromagnetic waves with a frequency, oscillation period, and wavelength ranging from 300 MHz–30 GHz, 3 × 10^−9^ s–33 × 10^−12^ s, and 1–10 mm, respectively. These waves have the ability to change the molecular rotation and ionic mobility of the medium without altering the sample. This rotation generates the adsorption and dissipation of energy in the medium, causing a generalized heating, forcing the rapid migration of all the active compounds from the solid-phase to the solvent-phase [7,13,14,15,16,17]

For this reason, the goal of this study was to define the optimal microwave-assisted extraction (MAE) conditions to render the bioactive compounds of annatto seeds without affecting their antioxidant and antimicrobial activities. The solvent concentration, pH, treatment time and solvent-to-seed ratio were taken as independent variables employing a surface response experimental design.

## 2. Materials and Methods

### 2.1. Materials

Sodium carbonate (lot a0594092339), anhydrous sodium acetate (lot 882032), absolute ethanol (lot k4958171742), potassium persulphate (lot k40707991048), methanol (lot l823009611), acetic acid glacial (lot k47109963539), acetone (lot k43912814243), sodium hydroxide (lot b0895598319), concentrated hydrochloric acid (lot k41017317016), Folin–Ciocalteu phenol reagent (lot hc43368401), Mueller–Hinton broth (lot b1223098542), tetrahydrofuran (lot dg643), and 2,4,6-tri-(2-pyridyl)-1,3,5-triazine (lot l58155738107) were obtained from Merck (Darmstadt, Germany). Dimethyl sulfoxide (lot 190260) was obtained from PanReac AppliChem (Barcelona, Spain). 2,2′-Azino-bis(3-ethylbenzothiazoline-6-sulfonic acid), 2, 2-diphenyl-1-picrylhydrazil (067k lot 1154), and diammonium salt (lot slbp9592v) were obtained from Sigma-Aldrich (St. Louis, MO, USA). (±)-6-Hydroxy-2,5,7,8-tetramethylchromane-2-carboxylic acid (Trolox) (97% lot stbb6668) was obtained from Aldrich Chem (St. Louis, MO, USA). Ferric chloride. 6H_2_O (lot 9n005099n) was obtained from Carlo Erba (Barcelona, Spain). 3-(4,5-Dimethylthiazol-2-yl)-2,5-diphenyltetrazolium bromide (MTT) (lot p31b064) was obtained from Alfa Aesar (Haverhill, MA, USA). Annatto seeds were purchased from a farmers market of Medellín city (Colombia), which were sun-dried until reaching a moisture content of 10.58 ± 0.98%.

### 2.2. Experimental Design

The MAE conditions were optimized using a Box–Behnken experimental design (BBD), employing the Design Expert Software^®^ Version 8.0.6 (Stat-Ease, MN, USA). The dependent variables for the two extraction methods are described in Table 1. From these conditions, 30 experimental runs were established and presented in a randomized pattern. The amount of total polyphenols determined by the Folin–Ciocalteu method, and the bixin (Bix) content were taken as independent variables [18]. 

**Figure 1:**
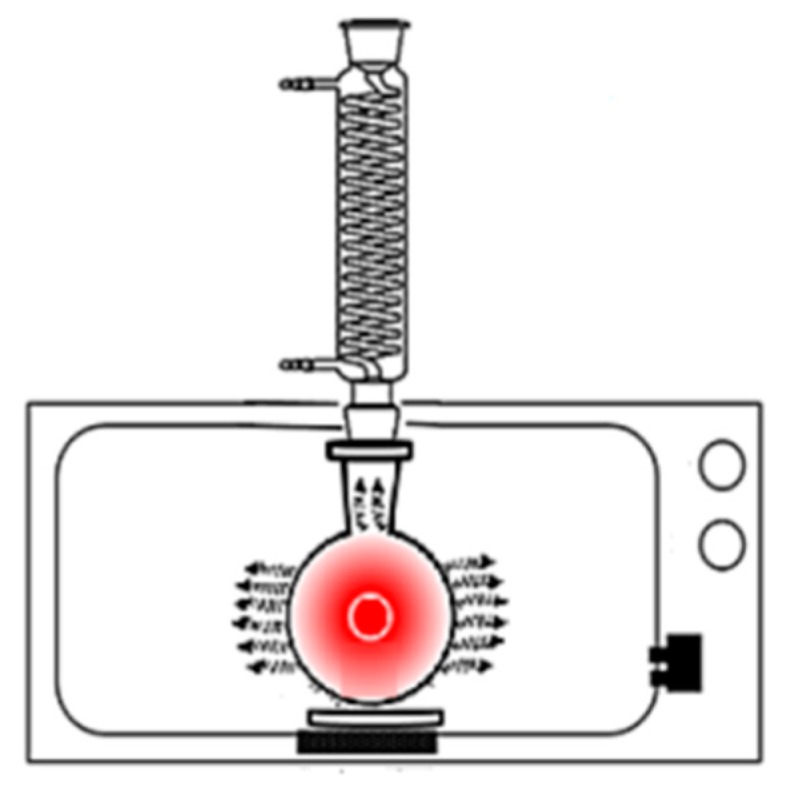
Schematics for microwave-assisted extraction (MAE) from annatto seeds.

The solvent pH at the concentrations determined by the BBD was adjusted with 0.1 M sodium hydroxide and acetic acid solutions. The microwave unit was operated at a frequency of 2450 MHz and power of 700 W (LG Ms-147xc, Seoul, South Korea), submitting the sample to 30-s cycles until reaching the desirable total time (Figure 1). In order to optimize the extraction conditions and investigate the effect of the independent variables the method of multiple regression of least squares was used. The coefficient of determination (r^2^) and the adjusted coefficient of determination (r^2^-adj model) were used as fitting parameters of the regression models. The experimental data were adjusted to the following polynomial equation:

Y= β_0_ + β_1_X_1_ + β_2_X_2_ + β_3_X_3_ + β_4_X_4_ + β_11_ X_1_X_1_ + β_22_ X_2_X_2_ + β_33_ X_3_X_3_ + β_44_ X_4_X_4_ + β_12_ X_1_X_2_ + β_13_ X_1_X_3_ + β_14_ X_1_X_4_ + β_23_ X_2_X_3_ + β_24_ X_2_X_4_ + β_34_ X_3_X_4_. (1)

where, Y represents the predicted response, β_0_, is the intercept of the model, β_1_, β_2_, β_3_, β_4_, β_11_, β_22_, β_33_, β_44_ and β_12_, β_13_, β_14_, β_23_, β_24_ and, β_34_ are linear and interaction coefficients, respectively, and X_1_, X_2_, X_3_, and, X_4_ are independent variables. The analysis of variance (ANOVA) was used to investigate the statistical significance of the independent variables from the models obtained (with a confidence level of 95%). The optimization of the extraction process was achieved by maximizing the extraction of polyphenol compounds and bixin having the same weight (weight of 1) and minimizing the solvent-to-seed ratio. The accuracy of the optimal conditions was determined with the desirability values of the dependent factors. The calculation of the relative and absolute errors was accomplished between the responses predicted by the model versus the ones obtained experimentally under optimal conditions.

### 2.3. Characterization of Optimal Annatto Seed Extracts

The optimal extract obtained by the MAE was compared with the one obtained by leaching. The latter was obtained with ethanol using at optimum concentration, pH, and seeds-to-solvent ratio for the MAE, with a continuous agitation for 48 h. The results are presented as means and standard deviation (SD). The analysis was performed using the Statgraphics^®^ Centurion XVI software (Madrid, Spain).

#### 2.3.1. Total Polyphenol Concentration

The total polyphenol concentration in the annatto seed extract was determined using the Folin–Ciocalteau method [19]. Briefly, 20 µL of sample were diluted in 1.58 µL of distilled water. Then, 100 µL of Folin–Ciocalteau reagent and 300 µL of 20% sodium carbonate were added and mixed. The absorbance of the colored complex generated was read after 1 h of storage under darkness. The maximum absorbance was read at 725 nm in a UV/VIS spectrophotometer (UV-1700, Shimadzu^®^, Kyoto, Japan). The experiments were performed in triplicate and the results are expressed as mg of gallic acid (GA) per grams of seeds (mg GA/g seed).

#### 2.3.2. Quantification of Bixin

Here, 100 µL of sample were added to 2 mL of tetrahydrofuran and diluted to 10 mL with acetone to obtain an absorbance of less than 0.15 at 487 nm. The concentration of bixin in the sample was determined from a calibration curve built in a spectrophotometer (UV-1700, Shimadzu^®^, Kyoto Japan) using the following equation (Equation (2)) [20]:

(2) $$Bixin\;\left(\%\right)=\frac{A\times 100\times V}{A_{1\mathrm{cm}}^{1\%}\times 100}$$

where

$A_{1\mathrm{cm}}^{1\%}$ = 3090 (1 g/100 mL)^−1^ × 1 cm^−1^ (specific absorptivity coefficient of bixin in acetone) [20];
A = Absorbance value of the sample; and
V = Dilution volume (mL) of the sample.

#### 2.3.3. Antioxidant Activity (ABTS^+^ Method)

The ABTS assay was performed following the method described by Contreras et al. 2011 [21]. Briefly, 100 μL of sample (diluted appropriately with water) were mixed with 1 mL of ABTS^+^ solution. The degraded color was read after 30 min at 730 nm using a spectrophotometer (UV-1700, Shimadzu^®^, Kyoto, Japan). A Trolox calibration curve was conducted for quantification purposes and the results are expressed as Trolox equivalents (TE) or μmol TE/L. 

#### 2.3.4. Ferric Reducing Antioxidant Power (FRAP)

The FRAP was measured as previously described by Benzie and Strain (1996) with modifications [22]. Briefly, 90 μL of deionized water were mixed with 30 μL of sample (diluted appropriately with water) and added to 900 μL of the FRAP reagent (pre-warmed at 37 °C). The sample was incubated for 30 min at 37 °C. Subsequently, the sample absorbance at 593 nm was measured using a spectrophotometer (UV-1700, Shimadzu^®^, Kyoto, Japan). A Trolox^®^ calibration curve was employed for quantification purposes and the results are expressed as μmol TE/L.

#### 2.3.5. Antioxidant Activity (DPPH Method)

The DPPH assay was performed as previously described [23]. Briefly, 2 mL of 0.5 mM DPPH reagent was mixed with 2 mL of methanol and 0.2 mL of sample (diluted appropriately with water). The absorbance peak at 517 nm was recorded after 30 min of incubation under darkness (UV-1700, Shimadzu^®^, Kyoto, Japan). A Trolox^®^ calibration curve was done for quantification purposes and the results are expressed as μmol TE/L.

#### 2.3.6. Antimicrobial Activity of Annatto Seed Extracts

The antibacterial activity was determined employing the colorimetric microdilution method with broth incubated with *Bacillus cereus* (ATCC 11778) and *Staphylococcus aureus* (ATCC 6538). Bacterial strains were incubated for 24 h at 37 °C in a Mueller–Hinton broth and adjusted to a final density of 10^6^ CFU/mL before inoculation. Samples were dissolved in dimethylsulfoxide (DMSO) to reach a final concentration of 4.096 mg/L. Double serial dilutions were made at a concentration ranging from 4 to 4096 mg/L. Then, 96-well microplates were prepared by adding 20 μL of sample on the respective dilution medium followed by addition of 220 μL of Mueller-Hinton broth. The last row containing only 220 μL of Mueller–Hinton broth and 10 μL of inoculum was used as a negative control. The final volume in each well was 250 μL. Once the samples were homogenized, they were incubated for 5 h at 37 °C. After incubation, 25 μL of 3-(4,5-dimethylthiazol-2-yl)-2,5-diphenyl-tetrazolium bromide (MTT) (Alfa Aesar, Germany), dissolved in DMSO (800 mg/L) were added to each well and incubated for 1 h to allow viable microorganisms to metabolize the yellow MTT dye in formazan. The minimum inhibitory concentration (MIC) was considered as the concentration of the first well that did not show any color change (from yellow to purple). The procedure was repeated three times for each microorganism and pH (4, 7, and 11) [1].

### 2.4. Statistical Analysis

The results are presented as means and standard deviation (SD), according to the normality of the data. The analysis was performed using the Statgraphics^®^ centurion XVI software (Madrid, Spain).

## 3. Results

### 3.1. Experimental Design

The experimental matrix was composed of 30 experimental runs. Experiments were performed in triplicate and the tabulated results from each experimental run are described in Table 2. The maximum values achieved in the extraction process were 4.36 ± 0.04 mg GA/g seed and 0.51 ± 0.01% of bixin. These values were obtained at 2.5 min time for MAE, pH of 11, and a solvent-to-seed ratio of 6:1. The only variation between the extractions corresponded to the solvent concentration used at a 50% for the maximum extraction of polyphenol compounds and 96% for the maximum extraction of bixin. 

ANOVA was used to evaluate the significance of the quadratic polynomial models. For each term in the models, a large *F*-value and a small *p*-value would imply a more significant effect on the respective response variable [24]. The ANOVA (Table 3) shows how factors such as treatment time, solvent concentration and the solvent-to-seed ratio had a statistically significant effect (*p* < 0.05) for the MAE. 

**Figure 2:**
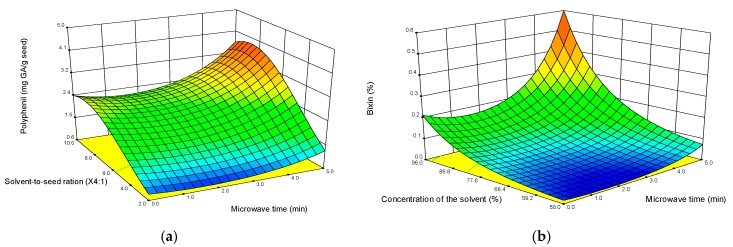
Response surface plots of solvent concentration (ethanol) and treatment conditions for: (**a**) polyphenol compounds and (**b**) bixin.

The response surface plots obtained from the polynomial equations are shown in Figure 2. All the models were subjected to an optimization process and the polynomial equations for the response variables are described as follows:

Ln (Polyphenols) = 0.70 + 0.03 × X_2_ − 0.26 × X_3_ + 0.53 × X_4_ + 0.26 × X_1_ − 0.42 × X_2_X_3_ + 0.18 × X_2_X_4_ − 0.41 × X_4_^2^ + 0.29 × X_1_^2^ (3)

Ln (Bixin) = − 0.59 − 1.12 × X_2_ − 0.04 × X_3_ + 0.76 × X_4_ − 0.15 × X_1_ + 0.04 × X_2_X_4_ − 0.01 × X_3_X_4_ + 0.06 × X_2_^2^ + 0.01 × X_4_^2^ + 0.10 × X_1_^2^ (4)

The response surfaces plots depict how the application of microwaves accelerates the process of extraction of the bioactive compounds. When a longer treatment time was applied the yield of bioactive compounds was larger. Once both dependent variables were optimized (polyphenol compounds and bixin), the estimated conditions for the MAE along with their relative error were as listed in Table 4. The absolute bias for the MAE process was high, and thus the experimental results obtained were greater than those predicted by the models.

The absolute error compares the results predicted by the optimized model to the experimental ones obtained under optimal conditions. It indicates that the bixin extraction models using MAE are more precise than those obtained for the extraction of polyphenol compounds.

### 3.2. Effect of the MAE on the Antimicrobial and Antioxidant Activities of the Extract

The antioxidant and antimicrobial properties of the extracts obtained at the optimal operation conditions of the MAE were compared to those obtained by leaching. Table 5 shows larger antimicrobial and antioxidant activities for the MAE than those obtained by leaching. This is explained by the higher content of polyphenol and bixin compounds present in MAE.

## 4. Discussion

The evaluation of the variables studied and optimized responses; microwave treatment time, pH, solvent concentration (%), and solvent-to-seed ratio (X_4_:1), showed a significant effect (*p* < 0.05) on the extraction of the polyphenol compounds and bixin from annatto seeds. Table 2 shows the variability of the results obtained in each experimental unit, with results varying from 0.53 to 4.36 mg GA/g seeds for polyphenol compounds and from 0.03 to 0.58% for bixin. This variability is confirmed by the ANOVA (Table 3), showing that all the independent variables studied had a greater influence in the extraction process for both metabolites.

The influence of these conditions on the extraction process is mainly due to (1) the increase in the mass transfer capacity and/or the extraction power of the system, (2) the affinity of the bioactive compounds towards the solvent, and (3) the effect of the heat generated on the micro-domains of the system [25]. By increasing the solvent-to-seed ratio, the saturation point of the system is favored increasing the mobility of the seeds in the agitated system, generating a greater mass transfer of the bioactive compounds to solvent [25]. The amount of solvent in the system not only affected the extraction capability of the system, but also had a statistically significant interaction effect (X_3_X_4_) (Table 3), improving the affinity of the compounds towards the extractive solvent resulting in an enhanced mass transfer process. Polyphenol compounds present in annatto extract (i.e., apigenin, hypolaetin, and the caffeic acid derivative) possess a larger polarity as compared to bixin, which is a highly conjugated long chain molecule, also known for having a very low solubility. Therefore, increasing the concentration of ethanol favors the release of bixin by having a higher solvent content to form hydrogen bonds, and in the case of polyphenol compounds the extraction is favored by its polarity [3,26,27].

On the other hand, the effect of pH was not significant in its linear term for the extraction of the polyphenol compounds and bixin. This result is opposite to that of Rubio-Senent and collaborators, who demonstrated that the activity and solubility of polyphenol compounds and bixin were affected by pH. Further, bixin, is a carotenoid having a highly conjugated structure and a carboxyl end-group. As a result, its solubility at neutral and alkaline pH was limited. Conversely, the polyphenol compounds possess an aromatic ring structure which is attached to hydroxyl groups or other planar rings. These molecules are slightly acidic and their solubility and activity are maximized at neutral and slightly acidic pH [28]. The insignificance of pH in the system can be attributed to the powerful effect of other independent variables studied which overshadowed its effect.

The microwave treatment time was significant (*p* < 0.05) since it increased the extraction power for the polyphenol compounds and bixin. However, polyphenol compounds were more sensitive to this extraction process than bixin (Figure 2a) since once the maximum point of extraction was reached, the content of polyphenols steadily decreased. This is explained by the heat generated during the microwave treatment. Thus, the longer the treatment, the larger the temperature reached in the system, causing solvent volatilization along with a partial degradation of the polyphenol compounds. Some authors have reported that the bioactivity of these secondary metabolites are affected by the extraction mechanism and heat treatments [7,29]. On the other hand, the optimal extraction conditions were found when a pH of 7, solvent concentration of 96%, solvent-to-seed ratio of 6:1, and a microwave time of 5 min were in place. These conditions rendered the largest yield of polyphenol and bixin compounds. It is then worthy to mention that the antioxidant and antimicrobial activities of the optimized extract were probably due to its largest content of polyphenol and bixin compounds, which in turn were responsible for their antioxidant and antimicrobial activities. These results coincide with those reported by Vasu et al. in 2010, which extracted only bixin from *B. orellana* seeds by applying microwaves for a time of 18 min as compared to a leaching extraction for 80 min. As a result, the bixin extraction raised from 8.2% to 16.3%. They also claimed that MAE is an alternative green technology and superior to conventional extraction for the extraction of bixin [12].

On the one hand, the antioxidant activity increased for the extract obtained by MAE, with a 5-and 10-fold increase of bixin and polyphenol compounds, respectively, as compared to the extract obtained by leaching. Moreover, the extract obtained by MAE presented antimicrobial activity against *B. cereus* and *S. aureus*. There were statistically significant differences (*p* < 0.05) for both activities between treatments, where MAE presented greater in vitro activities thanks to the high contents of polyphenols and antioxidant compounds. However, a direct proportional association was not appreciated between the increase of the bioactive compounds and the increments of the antimicrobial and antioxidant activities. Thus, the antioxidant activity was the most affected variable by the extraction method. The results of the antimicrobial and antioxidant activities of the lyophilized extract are comparable with those reported by some authors [1,12,30].

## 5. Conclusions

The MAE technique increased the yield of polyphenol compounds and bixin extracted from the annatto seeds, reducing energy consumption and increasing the antioxidant capability of extracts obtained. Despite the fact that the active compounds had a larger yield as compared to leaching, the antimicrobial activity did not present significant differences. 

## Figures and Tables

**Table 1:** Factor levels of the independent variables according to the Box–Behnken experimental design (BBD).

Independent Variables	Symbol	Coded Levels		
		−1	0	+1
Treatment time (min)	X_1_	0	2.5	5
pH	X_2_	4	7.5	11
Solvent concentration (ethanol) (%)	X_3_	50	73	96
Solvent-to-seed ratio (X_4_:1)	X_4_	2	6	10

**Table 2:** Experimental matrix and results obtained from the MAE.

Treatment Number	MAE (min)	pH	Solvent Concentration (Ethanol) (%)	Solvent-to-Seed Ratio (X_4_:1)	MAE	
					Polyphenols (mg GA/g Seed) *n* = 3	Bixin (%) *n* = 3
1	2.5	7.5	73.0	10.0	2.83 ± 0.04	0.13 ± 0.00
2	2.5	4.0	50.0	6.0	1.53 ± 0.22	0.03 ± 0.00
3	2.5	7.5	50.0	10.0	2.85 ± 0.19	0.05 ± 0.00
4	5.0	4.0	96.0	6.0	1.96 ± 0.06	0.33 ± 0.04
5	2.5	7.5	73.0	6.0	1.96 ± 0.15	0.05 ± 0.01
6	5.0	11.0	73.0	6.0	2.01 ± 0.08	0.13 ± 0.02
7	5.0	11.0	73.0	6.0	1.35 ± 0.05	0.32 ± 0.01
8	2.5	11.0	96.0	6.0	3.94 ± 0.00	0.51 ± 0.01
9	5.0	7.5	50.0	6.0	3.79± 0.07	0.04 ± 0.01
10	2.5	7.5	50.0	2.0	0.53 ± 0.08	0.27 ± 0.03
11	2.5	4.0	73.0	6.0	4.16 ± 0.24	0.19 ± 0.00
12	2.5	4.0	73.0	2.0	0.83 ± 0.06	0.12 ± 0.01
13	2.5	7.5	73.0	6.0	1.89 ± 0.14	0.05 ± 0.01
14	2.5	7.5	96.0	6.0	1.72 ± 0.15	0.19 ± 0.00
15	2.5	11.0	73.0	10.0	3.25 ± 0.57	0.04 ± 0.01
16	2.5	11.0	50.0	6.0	4.36 ± 0.04	0.03 ± 0.00
17	2.5	7.5	96.0	6.0	2.43 ± 0.01	0.58 ± 0.00
18	2.5	7.5	96.0	2.0	0.66 ± 0.01	0.38 ± 0.03
19	0.0	7.5	73.0	6.0	1.96 ± 0.08	0.06 ± 0.01
20	5.0	7.5	96.0	10.0	2.10 ± 0.01	0.13 ± 0.00
21	2.5	7.5	73.0	2.0	0.70 ± 0.01	0.07 ± 0.02
22	0.0	7.5	73.0	6.0	1.90 ± 0.23	0.04 ± 0.00
23	2.5	11.0	73.0	2.0	0.68 ± 0.03	0.17 ± 0.01
24	0.0	7.5	73.0	10.0	2.15 ± 0.16	0.10 ± 0.01
25	2.5	4.0	73.0	6.0	2.28 ± 0.01	0.12 ± 0.03
26	0.0	7.5	73.0	6.0	2.02 ± 0.03	0.08 ± 0.00
27	0.0	7.5	50.0	6.0	3.90 ± 0.30	0.08 ± 0.00
28	2.5	7.5	73.0	2.0	1.57 ± 0.30	0.25 ± 0.01
29	0.0	4.0	73.0	10.0	1.96 ± 0.15	0.33 ± 0.01
30	5.0	7.5	73.0	6.0	1.91 ± 0.18	0.06 ± 0.02

Values are expressed as mean ± standard deviation (*n* = 3). MAE: microwave-assisted extraction, GA: gallic acid.

**Table 3:** ANOVA for the response variables in the MAE.

Variable	Polyphenols (mg GA/g Seed)	Bixin (%)
	*p*-Value	*p*-Value
Model	<0.0001	<0.0001
X_1_, treatment time (min)	<0.0001	<0.0001
X_2_, pH	0.532	0.112
X_3_, solvent concentration (%)	<0.0001	<0.0001
X_4_, solvent-to-seed ratio (X4:1)	<0.0001	0.677
X_2_X_3_	0.001	˃0.050
X_2_X_4_	0.044	0.001
X_3_X_4_	˃0.050	0.004
X_2_X_2_	˃0.050	<0.0001
X_4_X_4_	<0.0001	˃0.050
X_1_X_1_	<0.0001	<0.0001
Lack of fit	0.0405	0.137
r^2^	0.931	0.914
r^2^-adj	0.901	0.875

**Table 4:** Predicted local maximum for the optimization of the MAE applying a Box–Behnken experimental design.

pH	Solvent (Ethanol) (%)	Solvent-to-Seed Ratio (X_4_:1)	Treatment Time (min)	Polyphenol (mg GA/g Seed)	Bixin (%)
7.00	96	5.95	5.00	2.69	0.58
Experimental result				3.08 ± 0.01	0.58 ± 0.02
Relative error				−0.39	0.00
Absolute error (%)				14.41	0.55

Values are expressed as mean ± standard deviation (*n* = 3).

**Table 5:** Comparison of the antioxidant and antimicrobial activities of annatto extracts obtained by MAE and leaching.

Extract		MAE	Leaching
Bixin	(%)	0.576 ^b^ ± 0.015	0.165 ^a^ ± 0.002
Polyphenols	(mg GA/g seed)	3.078 ^b^ ± 0.012	0.343 ^a^ ± 0.003
ABTS	(µM Trolox/L extract)	577 ^b^ ± 5	174 ^a^ ± 8
FRAP	(µM Trolox/L extract)	316 ^b^ ± 10	127 ^a^ ± 2
DPPH	(µM Trolox/L extract)	1043 ^b^ ± 50	811 ^a^ ± 5
*Bacillus cereus*	pH 11 (mg/L)	16 ^a^	128 ^b^
	pH 7 (mg/L)	16 ^a^	128 ^b^
	pH 4 (mg/L)	16 ^a^	128 ^b^
*Staphylococcus aureus*	pH 11 (mg/L)	8 ^a^	32 ^b^
	pH 7 (mg/L)	8 ^a^	32 ^b^
	pH 4 (mg/L)	8 ^a^	32 ^b^

Different superscript letters within row indicate significant differences (*p* < 0.05) according to LSD-Fisher; values are expressed as mean ± standard deviation (*n* = 3). ABTS: 2,2′-azino-bis(3-ethylbenzothiazoline-6-sulphonic acid), FRAP: Ferric Reducing Antioxidant Power, and DPPH: 2,2-Diphenyl-1-picrylhydrazyl
